# The Meaning of Establishing and Maintaining a Caring Relationship in the Prehospital Context as Experienced by Specialist Ambulance Nurses

**DOI:** 10.1111/scs.70301

**Published:** 2026-07-26

**Authors:** Andreas Rantala, Emelie Andreasson, Liza Dahlgren, Anna Forsberg

**Affiliations:** ^1^ Department of Health Sciences Lund University Lund Sweden; ^2^ Ambulance Service Department Region Skåne Helsingborg Sweden; ^3^ Ambulance Service Department Region Kalmar län Ålem Sweden; ^4^ Ambulance Service Department Region Kalmar län Kalmar Sweden; ^5^ Department of Cardiothoracic Surgery Skåne University Hospital Lund Sweden

**Keywords:** ambulance clinician, caring, emergency care, interaction, phenomenological hermeneutic, relationship, specialist ambulance nurse

## Abstract

**Aims and Objective:**

The aim of the study was to explore the meaning attributed by specialist ambulance nurses to the process of establishing and maintaining a caring relationship in the prehospital context.

**Background:**

Previous studies suggest that some patients may feel overlooked or not taken seriously in encounters with the ambulance service. Even brief caring relationships in prehospital care can support well‐being, strengthen patients' own resources, and help preserve dignity. However, less is known about how these relationships are experienced and interpreted by specialist ambulance nurses.

**Methods:**

Eleven specialist ambulance nurses were interviewed digitally in Sweden. Data were analysed using a phenomenological–hermeneutic method, developed by Lindseth and Norberg based on Ricoeur's epistemology, with the aim of interpreting the meaning of the lived experiences expressed in the interviews.

**Results:**

Specialist ambulance nurses' main concern was to achieve concordance, that is, a situation involving acceptance of the circumstances, consensus and concurrence in a state of harmony, leading to a caring ambulance clinician—patient encounter built on mutual trust and understanding. However, the participants also reported disruptive encounters with patients and their significant others involving resistance to the circumstances, disagreement, disbelief and a sense of frustration, resulting in a prehospital encounter built on mutual bias and misunderstanding.

**Conclusion:**

Caring relationships in the prehospital context are shaped by concordance and disruption, fostering relational connectedness or experiences of abandonment. Just care requires specialist ambulance nurses to recognise and challenge inherent biases, as otherwise health promotion may be overshadowed by the power to refuse care.

## 1 Introduction

Being a person experiencing illness, and possibly being in need of medical attention, entails a state of vulnerability [1] that can be understood as illness‐related suffering from a caring science perspective [2]. The ambulance service (AS) often represents the patients' first physical contact with the healthcare system, where ambulance clinicians (ACs) handle all sorts of illnesses or injuries and patients of all ages. However, previous studies indicate that patients do not always feel confirmed or taken seriously by the AS [3, 4, 5]. This may have implications, as irrespective of the type of illness, a caring relationship can generate feelings of well‐being and should be established in order to support patients' inherent health potential and preserve their dignity [6]. To ensure the provision of care that incorporates a caring relationship, the rationale behind this study is to understand how it can be established within the Swedish AS.

From a patient perspective, a caring relationship within the AS can evolve when the patient feels trust and security when interacting with the AC [7, 8]. Trust is strengthened when the AC is calm and respectful and involves the patient in the care. On the other hand, trust and the vulnerable care relationship can be compromised when the patient is excluded from a partnership and does not feel heard or seen. When no trust is established, there is a risk that the care provided by the AC will be questioned, with the patient viewing the AS as merely a transport system. How the patient is treated in an already vulnerable situation is of great importance for how the development of the care relationship [9].

Previous research indicates that ACs considered body language to be an important part of their professional self‐perception [10, 11, 12, 13]. Through body language, ambulance personnel can show respect for patients and build trust [12]. As described by Grechow et al. [14], language barriers can be something negatively affecting the relationship between patients and ACs. However, non‐verbal communication can be used as a common language to create a mutual understanding. Being present and available might create trust regardless of language barriers, where a friendly voice can provide a sense of security [12]. ACs strive to ensure that the encounter brings feelings of calm and peace to the patient [13] and creates a positive and tranquil atmosphere [15], hopefully leading to increased trust in their competence.

Patients' experiences of encounters with the AS reveal a broad spectrum of responses, where some interactions foster reassurance and involvement, whereas others give rise to frustration or emotional strain [3, 5, 16]. These divergent accounts underscore the fact that the prehospital care relationship is highly sensitive to situational demands and contextual influences, with communication, presence and clinicians' professional conduct interacting in ways that can either facilitate or hinder person‐centred relational work in everyday ambulance care [17]. Against this backdrop, a recognised need has emerged to further strengthen the professional role and conduct of ambulance personnel [18], highlighting the importance of interpreting ACs' lived experiences of the caring relationship. Thus, the aim of the study was to explore the meaning attributed by specialist ambulance nurses to the process of establishing and maintaining a caring relationship in the prehospital context.

### 1.1 Theoretical Framework

This study is grounded in caring science, understood as an autonomous discipline concerned with health, suffering, well‐being and caring [19]. Caring is in this context not reduced to tasks, procedures or medical interventions, but understood as an ethical and relational practice in which the patient's lifeworld, vulnerability, dignity and suffering are central [19, 20].

In this article, care refers to the broader professional and organisational practice of prehospital healthcare. Caring denotes the ethical and relational dimension through which the patient is encountered as a person, whereas nursing refers to the specialist ambulance nurse's professional practice including clinical knowledge, assessment, decision‐making, moral responsibility and interpersonal sensitivity [21, 22].

A caring encounter is more than a clinical meeting or an exchange of information. Shaped by presence, recognition, availability, uniqueness and mutuality, it may open a space in which patient and caregiver meet as human beings [23, 24]. However, the caring relationship is also ethically asymmetrical, as suffering may reduce the patient's capacity to act. This asymmetry can become ethically problematic if not balanced by reciprocity, respect and responsibility [25].

In the AS context, caring science research has highlighted the patient perspective, openness, sensitivity, caring relationships and ethical value conflicts in unpredictable encounters [13, 17, 22, 26]. Providing emergency care has also been described as an ethical response to the patient's lifeworld and existential needs, where professionals must balance patient advocacy with efficiency, time pressure and organisational demands [26, 27].

Against this background, caring relationships in the AS can be understood as ethically significant encounters in which specialist ambulance nurses (SANs) integrate expert nursing practice with trust, ethical presence, dialogue and responsiveness to the patient's lifeworld and suffering under unpredictable and time‐critical conditions [13, 17, 26].

### 1.2 Pre‐Understanding

The authors' pre‐understanding was informed by clinical experience within the ambulance service, teaching prehospital emergency care at university level, and familiarity with relevant research. In keeping with a phenomenological–hermeneutic approach, this pre‐understanding was acknowledged and continuously reflected upon throughout the interpretation of the material, moving between the interview texts, structural analysis and the emerging overall understanding [28]. During the analytical process, attention was given to how prior knowledge and experience might shape the interpretation, whereas remaining attentive to meanings arising from the data. The final interpretation was discussed among all four authors and agreed upon, although alternative readings were considered along the way.

## 2 Methods

### 2.1 Study Design

This study employed an explorative qualitative design grounded in phenomenological hermeneutics. The method, developed by Lindseth and Norberg [28, 29], based on Ricoeur's epistemology [17, 18], is intended to illuminate the meaning of lived experience [19]. In the present study, the phenomenon under investigation was the meaning of establishing and maintaining caring relationships in the AS context, as experienced by SANs. The qualitative method reporting adheres to the consolidated criteria for reporting qualitative research (COREQ) standard [20].

### 2.2 Setting

Sweden consists of 21 regions, each of which is responsible for the provision of healthcare including the AS and how it is staffed. In the Swedish region in which this study took place, ambulances must be crewed by at least one Registered Nurse (RN). The other team member can either be an Emergency Medical Technician (EMT) or a SAN, that is, holding a one‐year Master's degree. However, ambulance staff can also consist of nurses specialised in, for example, anaesthesia or emergency care. The person who has the highest medical degree, that is, RN or SAN, is responsible for the medical care during the meeting with a patient. The present study was conducted in a geographically large, but sparsely populated region in the south of Sweden, covering approximately 11,700 km^2^ and characterised by mixed urban and rural settlement patterns and potential long distances to hospital care. At the time of data collection, the AS handled approximately 37,000 missions annually, distributed across 15 ambulance stations.

### 2.3 Selection and Participants

A purposive sample was chosen to capture a variety of eligible participants in terms of clinical experience, working in urban or rural catchment areas, sex and age. The inclusion criterion was clinically operative SANs. Employees who met the inclusion criterion were approached at the 15 ambulance stations in the region. First, the operational manager gave permission to conduct the study. Subsequently, first‐line managers sent an e‐mail with information to employees who fell within the scope of the inclusion criterion. Participant information was provided to those who expressed an interest and included, among other things, the study's background, aim, voluntariness, and the possibility to withdraw from participation at any time. Eligible participants were encouraged to contact one of the authors [E.A. and L.D.] via e‐mail. After informed consent had been obtained, 11 SANs were recruited, eight women and three men, with a median age of 39.5 years, ranging between 29 and 56 years. Participants were recruited due to their experience of working as SANs within the regional AS. Previous Swedish AS research provides contextual support for understanding ambulance nursing as a nationally established but regionally organised specialist nursing context, with varying competence requirements and role expectations [30, 31]. The participants' demographics are presented in Table 1.

**TABLE 1:** Participants' demographics (*N* = 11).

	*n* (%)
*Sex*	
Men	3 (27)
Women	8 (73)
*Age (in years)*	
< 30	1 (9)
30–40	6 (55)
41–50	2 (18)
51–60	2 (18)
*Clinical experience in the ambulance service (in years)*	
1–5	1 (9)
6–10	7 (64)
11–15	3 (27)

### 2.4 Data Collection

All interviews were conducted during May and June 2022 by two of the authors [E.A. and L.D.]. A pilot interview that was not included in the study was conducted to ensure that the interview questions corresponded to the aim and to evaluate the content and interview technique [32]. The interviews were performed digitally [33] with the sound recorded via external equipment. The interviews started with the question ‘Could you please tell me about your experiences of caring relationships in the patient‐SAN encounter?’ The interviews were narrative and meaning‐oriented, focusing on the participants' lived experiences of caring relationships in concrete patient encounters. The opening question invited participants to narrate freely, and follow‐up questions were used to deepen the narratives, for example by asking them to describe what happened, how they experienced the encounters, what they perceived as important and how they understood their responsibility in the relationship with the patient. After 11 interviews, lasting for a median of 26 min, ranging between 19 and 46 min, the data content was considered rich and relevant to the study aim. The sample was considered adequate because all participants had lived experience of the phenomenon under study [28] and the interviews generated narratives with sufficient richness and variation to enable interpretation of the meaning of caring relationships in the AS [28, 29, 34]. The interviews were transcribed verbatim by two of the authors [E.A. and L.D.].

### 2.5 Data Analysis

Lindseth and Norberg have developed a phenomenological hermeneutic method [28, 29] based on Ricoeur's epistemology [17, 18]. This method was applied in order to study what was said in the interviews and to create an understanding of the meaning of the experiences, where the lived experience forms the starting point [28, 29].

The interviews were conducted, transcribed and analysed in Swedish, the language shared by both participants and the research team. Translation into English was undertaken only after the completion of the analysis, as part of the manuscript preparation [35]. When necessary, to preserve the meaning of participants' accounts and the nuances of the findings, the authors collaborated with a professional translator with subject‐specific experience. Translated quotations and key concepts were then carefully reviewed within the research team to ensure that the English wording remained close to the original Swedish meanings, whereas also being accessible to an international readership [36].

The phenomenological hermeneutic method comprises three steps, the first being *the naïve reading*, in which the interviews were read several times in order to become acquainted with the text and obtain an initial understanding [29]. In the second step, *the structural analysi*s, the text was separated into meaning units in accordance with the aim of the study. The meaning units were then brought together and sorted into themes and subthemes. In the final stage, *the comprehensive understanding*, the text was read again, reflecting on the themes concerning the meaning attributed by SANs to establishing and maintaining a caring relationship in the prehospital context. To enhance transparency, an example of the analysis process is presented in Table 2. All four authors participated in the data analysis process. Reflexive discussions were held throughout the analysis to ensure that the authors' pre‐understanding informed, but did not predetermine, the evolving interpretation.

**TABLE 2:** Example of the analysis process.

Meaning unit	Condensed meaning unit	Code	Subtheme	Main theme
When you arrive, you try to introduce yourself and explain who you are and why you are there	Introducing oneself and explaining one's professional role and purpose	Presenting oneself	Establishing contact and showing respect	Establishing a foundation of trust
Is there anything we can do to improve the situation? Can we help you? Can your relatives do something to make it better?	Asking open questions to explore how the patient and significant others can be supported	Asking open questions	Involving the patient and significant others	Maintaining the caring encounter

### 2.6 Ethical Considerations

The ethical code of conduct was followed, and we adhered to the ethical guidelines issued by the Swedish Research Council. Consent, confidentiality, utility and information were considered in line with the Declaration of Helsinki [37], as well as the Swedish ethical protocol and legislation [38]. All participants received both verbal and written information about the study aim, including the fact that participation was voluntary and that they could withdraw from the study at any time without negative consequences for themselves. The participants were also informed that their responses could not be traced back to them. Written informed consent was obtained from the participants before all interviews. The study was vetted by the Swedish Ethical Review Authority, who provided an advisory approval (Reference No. 2021‐03981).

## 3 Results

The *naïve* understanding revealed that the main concern of the Swedish SANs was to achieve concordance between themselves and the patients regarding the outcome of the care situation, for example, being discharged at the scene or transported to the hospital Accident & Emergency (A&E) department. Therefore, the first thematic structural analysis (Table 3) covers four themes illustrating the meaning of concordance, that is, a situation involving acceptance of the circumstances, consensus and concurrence in a state of harmony, leading to a caring AC—patient encounter built on mutual trust and understanding.

**TABLE 3:** Structural analysis.

Sub themes	Main themes
Structural analysis of the meaning of concordance when establishing ambulance service encounters	
Establishing contact and showing respect Confirming and taking seriously Being present and honest	Establishing a foundation of trust
Listening to the patient's narrative Being present in a caring space Using both verbal and non‐verbal communication	Practicing caring communication
Providing advice and information Guiding throughout the whole pre‐hospital process	Piloting as a deliberative caring act
Involving the patient and significant others Standing by until the assignment is over	Maintaining the caring encounter
Structural analysis of the meaning of disruption resulting in possible uncaring ambulance service encounters	
Feeling biased and frustrated Focusing mainly on personal chemistry Feeling questioned by the patient	Not making an effort to establish trust
Questioning the patient's will and integrity Questioning the patient's experiences and motives Blaming the patient for the failed encounter	Casting doubt on the patient
Failing to recognise the patient's suffering Anticipating gratitude in return for care Maintaining power in the caring relationship	Exercising paternalistic power

When concordance was achieved a potentially caring prehospital encounter took place. However, the participants also described disruptive encounters with patients and their significant others, which mainly involved those who frequently phoned for the AS or persons with substance abuse and addiction problems. Thus, a second thematic structural analysis was performed (Table 3) covering three themes illustrating the meaning of disruption such as resisting the circumstances, disagreement, disbelief, and a sense of frustration, which led to a prehospital encounter built on mutual bias and misunderstanding. During such an encounter, the perspectives and expectations of the patients and SANs differed profoundly, resulting in a disruptive and potentially uncaring prehospital encounter.

### 3.1 The Structural Analysis of the Meaning of Concordance

#### 3.1.1 Establishing a Foundation of Trust

The key issue for the SANs was establishing a foundation of trust, not only for the present situation, but to facilitate the next step and future encounters with other nurses, for example, in the A&E or at the primary healthcare centre. The mission was to establish a sense of trust in the overall healthcare organisation:

If I can establish a strong sense of trust in us, perhaps I can also transfer that trust to the nurses in the A&E and the physicians she will meet. (Informant no. 2)

The intention behind this extensive trust creation effort was to establish contact by showing respect and trying to understand the patient's behaviour as well as life world and social context. Confirming and taking the patient's symptoms and suffering seriously was essential. Visiting patients in their own homes had a specific meaning for the SANs in terms of the inherent power imbalance. Being present and honest when assessing, judging and communicating the decision about further caring actions was considered important. They emphasised that the patient might not have chosen to summon the ambulance. The trust involved in being invited into the patient's home was viewed as a possibility that must be treasured. Concordance was the goal, which was achieved through eye contact, touch, listening, non‐threatening body posture, being humble, an invitation to participate, confirmation, explanations and sometimes persuasion. The SANs perceived that their ability to show respect and humility when faced with the patient's vulnerability and limited integrity affected the whole encounter.

#### 3.1.2 Practicing Caring Communication

To achieve concordance, the SANs made great efforts to listen to the patient's narrative and to practice caring communication as a foundation for participation. By being open and non‐confrontational with no threatening body language, the SANs communicated that their mission is to help, where fulfilling that mission is achieved by listening to the patient's narrative. The SANs were highly aware of the importance of being present with the patient by creating a caring space in the patient's home, to which both the patient and their significant others were invited. In this way, they communicated their wish to serve and protect the patient. They acknowledged the moral responsibility involved in being close to someone who is vulnerable and exposed in their own home:

It is a great responsibility, both medically and interpersonally. You enter someone's home and immediately you are close to that person. You rapidly invade their space. So, you need to be careful and at the same time attentive and assess the situation. (Informant no. 9)

The SANs used both verbal and non‐verbal communication. They assessed the patient's facial and other non‐verbal expressions. The encounter was viewed as mutual interplay in a shared situation. Not all patients are conscious or lucid. In such cases the communication becomes sharper and clearer in order to break through the ‘filter’, but always with the utmost respect and an effort to avoid coercion.

#### 3.1.3 Piloting as a Deliberative Caring Act

Piloting was triggered by a will to facilitate patient participation, coherence and ultimately concordance. Providing advice and information was part of preparing the patient for the next step in the care process. It was important not to confuse the patient or use technical language:

There is always a risk that we do what we are used to doing, checking the vital signs without explaining why. But that doesn't work for the patient. To participate in their care, they need to know why and what we find. And then you have a good relationship from the start and that benefits the whole process. (Informant no. 7)

The piloting lasted throughout the whole encounter, showing that the patient was viewed as a valid partner. The encounter was considered successful when the SAN could meet the patient's expectations. If that was not possible it was important to be honest about the reasons for disappointing the patient.

#### 3.1.4 Maintaining the Caring Encounter

The effort to maintain the caring encounter during conveyance to the hospital required continuously involving the patient and significant others. A concrete way was to validate one's assessment and judgement with the patient while recording the patient's symptoms and vital signs. Another way was to show persistent concern during the transport and ensure that the patient was prepared to be left at the A&E. As one participant explained:

You need to show that you really care all the way from the first contact with the patient, during the transport to the hospital until you leave him or her, for example in the A&E, and that you transfer your judgement of the patient's situation to the next professional. (Informant no. 6)

Together, these actions illustrate how maintaining the caring encounter demanded a continuous and intentional presence, possibly throughout the entire care trajectory.

### 3.2 The Structural Analysis of the Meaning of Disruption

#### 3.2.1 Not Making an Effort to Establish Trust

When failing to establish trust the encounter is mainly based on personal chemistry and the SANs' impression of the atmosphere in the room. In such situations, SANs can be clearly biased by their attitude towards frequent AS users. The frustration about the phenomenon of frequent users presumably abusing the AS is projected onto the patient. In these situations, SANs have the impression that the patient disrespects their judgement and competence. When feeling questioned by the patient or significant others, SANs want to manifest experience and competence, resulting in the patient being left at the scene and not deemed to need ambulance transport. The interpersonal dynamic includes a manifestation of power, that is, ‘if you don't respect my judgement, you won't be transported to the hospital’. SANs blame the patient, who is viewed as spoiled, impatient and misinformed:

I don't think he [the patient] could stand that a woman younger than himself denied his wishes. In that situation he didn't respect me at all… and then I got frustrated sort of…when you realize that the person is not particularly ill you get really tired…We are expected to put up with everything…and at some point, I am only human. This is my job, and I am not supposed to put up with everything for goodness' sake. Today people only expect to get what they want, they don't care about us. So, my patience is slightly gone. I am tired of arguing all the time. (Informant no. 10)

These situations illustrate how strained encounters can evoke defensive reactions in the SAN, reinforcing power dynamics and weakening the foundation for a caring interaction.

#### 3.2.2 Casting Doubt on the Patient

During disruptive encounters with patients, the SANs adopted a judgmental approach, questioning the patient's will and integrity as well as their experiences and motives for calling an ambulance. The SANs reflected a great deal on the different ways in which patients tried to misuse the AS and the overall healthcare system. In these situations, they tried to defend the system and acted as gatekeepers to protect the A&E or other primary healthcare centres from the patient. They also blamed the patient for the failed encounter:

I can go to someone who I know misuses the healthcare system and I can sit in the car and say to my partner, here we go again. Of course, I am annoyed, even if as an ambulance nurse I don't want to feel that way. But I am only human…It is more difficult to establish a caring encounter with the frequent users because my impression is that they abuse the AS. (Informant no. 4)

These accounts show how frustration, moral judgement and perceptions of misuse can shift the interaction from a caring stance to a defensive and distancing posture.

#### 3.2.3 Exercising Paternalistic Power

When disruptive care encounters occur there is a battle between the patient and SAN to see who will give in, thus the focus is not on symptoms, suffering or nursing. This specifically concerns frequent ambulance users, who the SANs consider are in need of a reality check due to the perception that by presenting often and receiving unnecessary care they are a burden to the healthcare system. The SAN believes that the patient's disappointment about being left at home instead of conveyed to hospital is the cause of the poor encounter and blames the patient for having the wrong expectations. The SANs excuse themselves by claiming to be only human and lacking patience.

Furthermore, SANs expect the patient to show gratitude and be pleasant and friendly during long journeys to the hospital, believing that gratitude is an indication of a good caring encounter. When the patient is silent and thus boring, the whole transport becomes an ordeal. The entire focus remains on the SAN's patience, job satisfaction, and approach as well as what the patient can offer in terms of interactional skills.

Inherent in the disruptive encounters is the SAN's need to maintain power. Persons with established or known substance abuse are judged beforehand. On their way to the patient, SANs complain to colleagues that it is not necessary to respond to this call. The point of departure is the view that the patient is lying and being manipulative, where the goal is to refuse to convey the patient, who then becomes agitated. The SAN's line of argument is that if you give your little finger, the patient will take your whole hand and thus no care is provided. The patient is not taken seriously and the SAN's perceived power is maintained:

You know from the start that you are going on what we call a false alarm. The guy wants help with something that does not concern us. He says that he is in pain or has some other complaints. He simply lies about his symptoms to get us to visit him. And we still must go there… but it is not easy to keep a professional attitude. Of course, you let off steam a lot on the way and then back to the station, but we try to keep it in the car. He [the patient] is only focused on getting what he wants and when that doesn't happen, he becomes aggressive. So, it doesn't matter what we do. Simply not give him anything. If you give him your little finger, he tries to take your whole hand. (Informant no. 3)

Thus, this sub‐theme shows how the SANs' assumptions and authority came to dominate these encounters, positioning the patient as someone whose needs and explanations could be overridden.

### 3.3 Comprehensive Understanding

As expected, prehospital care involves both potentially caring and uncaring encounters. The new and comprehensive understanding is that encounters deemed as non‐urgent by the AS and SANs are characterised by either concordance or disruption. The SAN strives for concordance in every encounter with patients considered worthy of such an effort and uses the steps in the deliberative nursing process [39] in order to achieve a state where both parties involved accept the circumstances, reach consensus and concurrence in an atmosphere of harmony built on mutual trust and understanding. However, when distrust and suspicion precede the contact, the patient–SAN encounter is disrupted by bias, unmet expectations and disappointments. The SAN feels questioned and disrespected by the patient and in turn questions and disrespects the patient. A vicious circle is created filled with resistance to the circumstances, disagreement, disbelief and frustration, leading to a prehospital encounter ending up in a blame game where the patient is accused of causing the poor encounter due to unrealistic expectations. Thus, patients are very dependent on the SAN's judgement that they are worthy of being taken seriously, respected and receiving care. Concordance can mainly be evidenced by the patient's expressed gratitude and satisfaction with care before the SAN leaves the scene or during conveyance to the A&E department, where trust is the essence of the whole patient experience [8, 40]. Although the SAN wants to minimise the patient's state of impotency, in cases of disruption this impotency is increased by the SAN manifesting the power to reject the patient. Thus, in the first case the patient's home becomes a safe caring space, whereas during the disruptive encounter the home becomes an unsafe caring space.

## 4 Discussion

The findings show that the main concern of the SANs was to achieve concordance with patients regarding the outcome of the care situation. Concordance was experienced as acceptance, consensus and concurrence, leading to a potentially caring prehospital encounter built on mutual trust and understanding. In contrast, disruption was characterised by resistance, disagreement, disbelief and frustration, resulting in a potentially uncaring encounter shaped by mutual bias and misunderstanding.

Interpreted through a caring science perspective, these findings show that caring relationships in the AS were experienced as an ethical and relational responsibility rather than merely as a professional task or procedural component of care [19, 22, 26]. The movement between concordance and disruption illustrates how the caring encounter may either support or threaten the patient's lifeworld, vulnerability and dignity. When SANs responded with presence, dialogue and sensitivity to the patient's situation, the encounter could become a caring space shaped by trust, reciprocity and mutual understanding. Conversely, when distrust, bias and frustration dominated, the asymmetrical relationship risked becoming ethically problematic and potentially uncaring, as the patient's vulnerability could be reinforced rather than alleviated [24, 25]. This was reflected in the participants' descriptions of serving as a healthcare professional as including a responsibility to establish and maintain caring relationships. This responsibility involved showing consideration for the patient's entire life situation and needs [7], regardless of whether the condition was assessed as urgent or non‐urgent. It was experienced as more than merely performing their work based on a job description and guidelines, particularly in light of patients' trust that the AS is a just institution that attends to each patient in their unique context. In this sense, the findings illustrate how nursing in the AS involves integrating clinical assessment and decision‐making with moral responsibility and interpersonal sensitivity [22]. The participants' descriptions of responsibility may be understood in light of the AS being a just and fair institution [41], where both patients and healthcare professionals are implicitly oriented towards justice on a fundamental level. There are many obstacles to ensuring that the patient has an impression of being fairly treated, whereas being treated unfairly is something that is difficult to express in words [41]. Fulfilling that responsibility and seeing the person's needs regardless of experienced illness or perceived disease is a unique mission for the AS and SANs. Thus, the participants in the present study acknowledged that the SANs' main task is to ease the patient's suffering with available resources, along with a wish for all patients to receive an adequate medical assessment. To balance the two, it is necessary for the SAN to create conditions for a good caring relationship by seeing the person behind the illness and adopting an ontological approach to the caring situation [42, 43, 44]. However, the participants' accounts also suggested that this mission was not consistently extended to all patients, particularly those implicitly regarded as less deserving of AS resources.

The participants emphasised the importance of attentiveness in patient encounters, particularly in situations characterised by unpredictability and emotional complexity. Such attentiveness can be understood as an expression of lifeworld sensitivity, where the SANs remain open to the patient as a person whose suffering, vulnerability and need for reassurance are situated in a particular life context. Encounters and interactions with patients may evoke emotions within SANs. Exchanging feelings within the dyadic ambulance team can enable awareness of how the various team members react to different patients and situations, thereby being reflective and aware of possible shortcomings. Before entering the scene, it is important to take a few deep breaths, that is, enabling confidence to face the often unpredictable environment and remain attentive to one's responsibility for the patient [45]. This illustrates how a caring relational space may be fostered when the SAN conveys a sense of ‘we are in this together’ with the grateful patient who exhibits relevant symptoms or signs of illness. Conversely, when patients, such as frequent attenders, are met with distancing attitudes, the encounter risks being experienced as ‘you are in this alone’. There are different types of patient‐caregiver relationships. In concordance, the relationship may be understood as caring, or at least potentially caring, due to the fact that it carries central dimensions of the caring encounter: presence, recognition, uniqueness and mutuality. In disruption, the relationship moves away from these dimensions. Instead of being merely an uncaring encounter, it may be understood as a relationship in which the caring encounter is absent or severely compromised. In such situations, the SAN's professional authority becomes dominant, giving rise to a paternalistic approach [23, 24]. The communication patterns also differ between concordance and disruption, which can be described as caring and uncaring conversations, respectively [25]. When in concordance the SAN is present in the moment, not only physically but by being in a communion with the patient. On the other hand, when in disruption the SAN is physically present, but mentally absent, that is, being there in the room but not with the patient.

It has been demonstrated that a person's background and experience may shape their impression of places and affect their opportunities and activities [46]. In nursing geography, it is argued that there is a dynamic between nursing, health and place [47]. A spatial perspective may be a useful way to think about the depth of the nurse–patient relationship, as the relationship itself is spatially determined. It has been stated that place is also relevant to moral agency and activity by restricting or enhancing care and justice, thus affecting both personal and power relationships [48]. In this sense, place and proximity may support or restrict the SAN's ability to respond to the patient's lifeworld, vulnerability and suffering.

The participants described that the need for ambulance transport to the A&E department could be a source of conflict due to a notion among patients of a constant lack of resources, for example, ambulances. According to the participants, this entails a risk that when the AC's assessment results in a decision not to transport them to hospital, they may believe that they do not need to seek other medical attention [49]. Differences of opinion do not need to result in a conflict between the patient and the SAN. If patients receive adequate and clear information, they can feel satisfied even if their expectations regarding care are not fulfilled [50]. When SANs are empathic and take the patient seriously, the patient feels competent and respected [5, 49]. A patient who obtains a thorough assessment and receives relevant, understandable advice and help feels safe remaining at home instead of feeling insecure and rejected [5]. Our understanding is that it is possible to achieve concordance and a caring encounter by means of a deliberative nursing process [39] and a person‐centred ethical approach [41, 51]. From the caring science perspective used in this study, concordance is therefore not merely agreement about transport or treatment, but involves whether the patient is encountered as a person whose lifeworld, concerns and vulnerability are taken seriously [20] in the caring relationship by means of caring communication [25]. However, this can only occur if the SAN is aware of and challenges the inherent bias involved in the encounter with, for example, frequent attenders and persons with a substance abuse problem, patient groups previously shown to be considered undeserving of nursing interventions by SANs [22].

The participants' accounts of proximity, distancing and moral tension in encounters can also be illuminated through a spatial perspective on nursing. Nursing has spatial features and relies on proximity, where physical proximity facilitates closeness when nurses touch and physically act on behalf of patients. This may be considered a prerequisite for achieving concordance and extensively practised by SANs. Moral proximity, which concerns acting and safeguarding patients' interests, is hindered by the conscious bias among the SANs towards certain groups of patients, thus ruining the nurse–patient spatial dynamic. This may be interpreted as a form of organised suffering caused by care [52], in this case the prehospital AS system, and undermines the solid foundation of nursing, which involves health promotion within a caring relationship regardless of the patients' circumstances. From a caring science perspective, such distancing may disrupt the caring relationship. Care may still be provided in a technical or organisational sense, whereas caring is weakened or absent. This risks moving the encounter towards an uncaring form of care, where the patient's lifeworld, vulnerability and suffering are insufficiently recognised [21, 22, 53]. Consequently, the findings indicate that the meaning of caring relationships in the ambulance service lies in the SAN's ability to integrate expert nursing practice with ethical presence, dialogue and responsiveness to the patient's suffering. This aligns with the interpersonal process of caring in nursing involving expert nursing practice and interpersonal sensitivity [21]. Nursing in the AS is grounded in the establishment of caring relationships [22], supported by previous ambulance and emergency care research emphasising trust, dignity, ethical responsibility and responsiveness to the patient's lifeworld under time‐pressured conditions [17, 27, 53].

### 4.1 Methodological Considerations

When considering rigour, trustworthiness was guided by the criteria described by Lincoln and Guba [54]. Credibility was supported through open‐ended interviews with follow‐up questions that allowed participants to elaborate on their experiences. Although the interviews were relatively brief, participants showed a focused engagement with the topic, likely supported by receiving study information in advance and expressing a genuine interest in the subject matter. Dependability was supported by a consistent approach to data collection. All interviews were conducted digitally, which still allowed observation of non‐verbal cues, whereas audio was recorded using external equipment to ensure good sound quality. Confirmability was addressed through reflexive awareness of the researchers' pre‐understanding. Three of the authors have clinical experience as ACs, which may have influenced interpretation. However, none had prior relationships with the participants. Ongoing dialogue with the last author, who has no clinical background in this context but extensive qualitative expertise, supported reflexivity and helped reduce the risk of over‐interpretation. Transferability may be limited, as participants were recruited from a single region in Sweden. At the same time, there was variation in age, sex and professional experience. Organisational culture and management structures may influence how caring relationships are enacted, which should be considered when reflecting on the applicability of the findings to other contexts. This also aligns with a phenomenological hermeneutic understanding of validity, where the aim is not statistical generalisation but meanings that may be recognisable in similar contexts [28].

## 5 Conclusions and Implications

In conclusion, this study deepens the understanding of how caring relationships in the prehospital context are shaped by both concordance and disruption, revealing encounters that may be experienced as either caring or uncaring. Concordance fosters a sense of closeness grounded in partnership and caring communication, whereas disruption stems from uncaring communication and generates distance that may leave patients with experiences of abandonment. Ensuring just care requires SANs to confront their inherent biases, as the findings demonstrate how health promotion may otherwise be overshadowed by the exercise of the power to refuse the patient who needs care.

The findings highlight the importance of reflective practices that enable SANs to recognise how caring and uncaring tendencies emerge within prehospital encounters. Structured ethical and relational reflection may strengthen clinicians' capacity to preserve closeness and mitigate experiences of abandonment in vulnerable situations. This does not imply that SANs lack formal caring science education. Instead, the findings point to the need for continued reflection on how caring science knowledge is enacted in practice, especially when professional authority, organisational expectations and patient needs are difficult to reconcile. Integrating caring science within SAN education, as well as in continuing professional development, may therefore deepen the understanding of caring as an ethical and relational practice that is inseparable from clinical judgement and medical responsibility. Future research should explore how organisational conditions enable or constrain the realisation of caring relationships in the AS.

## Author Contributions

Conceptualization: A.R. Data curation: A.R., E.A. and L.D. Formal analysis: A.R. and A.F. Investigation: E.A. and L.D. Methodology: A.R., E.H. and L.D. Project administration: A.R. Resources: A.R. Supervision: A.R. Validation: A.R., E.A., L.D. and A.F. Visualisation: A.R., E.A. and L.D. Writing – original draft; A.R. and A.F. Writing – review and editing: A.R., E.A., L.D. and A.F. All authors read and approved the final manuscript.

## Funding

Open access funding provided by Lund University. This study received no external funding.

## Ethics Statement

The ethical code of conduct was followed and we adhered to the ethical guidelines issued by the Swedish Research Council. Consent, confidentiality, utility and information were considered in line with the Declaration of Helsinki [23], as well as the Swedish ethical protocol and legislation. All participants received both verbal and written information about the study aim, including the fact that participation was voluntary and that they could withdraw from the study at any time without negative consequences for themselves. The participants were also informed that their responses could not be traced back to them. Written informed consent was obtained from the participants before all interviews. The study was vetted by the Swedish Ethical Review Authority, who provided an advisory approval (Reference No. 2021‐03981).

## Consent

The authors have nothing to report.

## Conflicts of Interest

The authors declare no conflicts of interest.

## Data Availability

The datasets generated and/or analysed during the present study are not publicly available due to participants' confidentiality but are available from the corresponding author on reasonable request.
